# Clinical outcomes of vacuum-dehydrated amniotic membrane (Omnigen) mounted on contact lens (Omnilenz) in eyes with acute chemical eye injuries

**DOI:** 10.1007/s00417-023-06151-9

**Published:** 2023-06-26

**Authors:** Nancy M. Lotfy, Salah Al Rashidi, Sherein M. Hagras

**Affiliations:** 1 Department of Ophthalmology, Faculty of Medicine, Cairo University, Al-Saray Street, El Manial, Cairo, 11956 Egypt; 2 Farwaniya Hospital, Al Farwaniyah, Kuwait; 3 Faculty of Medicine, Mansoura University, Mansoura, Egypt

**Keywords:** Amniotic membrane, Chemical eye injury, Omnilenz, Epithelial defect, Limbal ischemia

## Abstract

**Background:**

Omnigen is a vacuum-dehydrated amniotic membrane transplant. It can be delivered to the eye pre-mounted on a special bandage contact lens (Omnilenz) that enables its application without the need for sutures or glue; the aim of this study is to evaluate the short-term clinical outcomes of Omnilenz-Omnigen complex in eyes with acute chemical injury (CEI).

**Methods:**

A prospective interventional study included patients with different grades of acute CEI attending the casualty between July 2021 and November 2022. All patients received first aid measures followed by the application of Omnilenz-Omnigen within the first 2 days. Patients were followed up for at least 1 month. Primary outcomes include epithelial defect and limbal ischemia. Secondary outcomes include best-corrected visual acuity (BCVA) and tolerability.

**Results:**

The study included 23 eyes of 21 patients with acute CEI mostly due to alcohol (34.8%). After the 1^st^ application, the size of the epithelial defect showed a statistically significant reduction (*p* = 0.016) with improvement in BCVA (*p* < 0.001). Restoration of the limbal vascularity was obtained in 56.5% of the eyes. Repeated application of Omnilenz was required in 5 eyes (21.7%). The size of the epithelial defect was reduced after the second application (*p* = 0.504) with improved BCVA (*p* = 0.185). After 1^st^ month, complete epithelial healing was achieved in all the eyes. Mild limbal ischemia persists in 3 (13%) of the eyes. Final BCVA showed statistically significant improvement (*p* < 0.001). None of the patients develops any serious complications.

**Conclusion:**

Omnilenz proved to be easy to apply and well tolerated by patients, with promising clinical outcomes.

**Supplementary Information:**

The online version contains supplementary material available at 10.1007/s00417-023-06151-9.

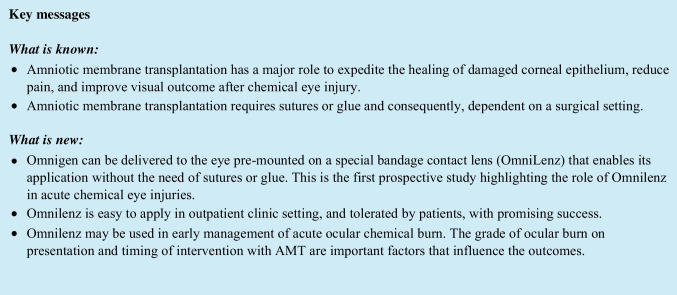

## Introduction

Chemical eye injury (CEI) represents 10–22% of all ocular trauma and is the second most common cause of work-based eye injuries comprising 12%, coming after ocular foreign body injuries which represent the vastness (43%) [1]. The severity of the ocular surface chemical injury is determined by the causative agent, the duration of contact, the treatment given, and the time from injury to initiation of treatment. These factors influence the depth of penetration of the chemical agent and the wound healing response [2].

For decades, amniotic membrane transplantation (AMT) had been used either as a graft, which can provide a basement membrane for epithelialization, or as a patch, where it acts as a biological bandage contact lens [3]. Studies have shown that AMT was able to expedite the healing of damaged corneal epithelium, reduce pain, and improve visual outcomes after CEI. Typically, AMT requires sutures or glue and is, therefore, dependent on a surgical setting [4].

Technologies allowed to apply the amniotic membrane to the ocular surface without the need for sutures [5]. An innovative technique allows applying of vacuum-dehydrated AM (Omnigen) using a customized contact lens (Omnilenz). Omnilenz can be applied simply within a few minutes in an outpatient clinic [6]. Currently, there are a few published reports regarding the use of Omnilenz in CEI. The aim of the current study is to evaluate the clinical outcomes of Omnilenz-Omnigen complex application after a chemical eye injury.

## Methodology

A prospective interventional study included patients with acute chemical eye injury attending the casualty department of Farwaniya Hospital, Kuwait, during the period from July 2021 to November 2022. The study follows the tenets of the Declaration of Helsinki. The study was approved by the ethical committee of Kuwait and registered in clinical trials.gov (NCT05618522). Written informed consent was obtained from all the patients prior to the treatment. Adult patients older than 18 years old with different grades of acute chemical eye injuries were included in the study. Patients with chronic CEI, absent or shallow fornices, and\or symblepharon interfering with contact lens placement were excluded.

First-aid therapy consists of extensive irrigation of the injured eye with normal saline (0.9%) at room temperature. Usually, this therapy was initiated in the casualty room, before referral to the corneal unit. In all cases, irrigation was continued until normalization of pH was confirmed. Superior and inferior conjunctival fornices were also examined, and any particulates were removed during the wash.

Baseline ocular examination included best corrected visual acuity (BCVA) using Snellen’s chart converted to Log MAR for statistical analysis. Counting fingers were graded as Log MAR 2.0, and hand motions were graded as Log MAR 2.3. Identifying the stage of a chemical eye burn was done according to Roper-Hall [7]. Limbal ischemia was graded as mild if affecting <1/3 limbus, moderate from 1/3 to 1/2 limbus, and severe if affecting >1/2 of the limbus. Cornea epithelial defects were described in terms of the percentage of the total corneal surface area. Examination of lids (for blepharitis, edema) and conjunctiva surface epithelial defects (in clock hours) with routine anterior segment examination for anterior chamber reaction, iris, pupil, and the lens was done using a slit lamp. Fundus examination was done by indirect ophthalmoscope if the media was clear or b-scan ultrasonography in cases of opaque media. At each follow-up visit, patients were asked a simple questionnaire about how they feel regarding the contact lens. Answers were either 1-tolerable, 2-feeling discomfort, or 3-needs patching.

All patients received oral ascorbic acid 1 g twice daily. Topical treatments included hourly topical preservative-free prednisolone 1%, antibiotic (ofloxacin 0.3% three times daily), and cycloplegics (cyclopentolate 1% three times daily). Patients received frequent artificial tear substitutes (hyaluronic acid 0.15%). In cases complicated by raised intraocular pressure, topical antiglaucoma drops were commenced usually in the form of a combination of beta-blockers and topical carbonic anhydrase inhibitor, followed by the use of systemic acetazolamide, if not controlled.

Omnigen of size 16 mm was placed on Omnilenz (www.nu-vision.co.uk) [6] and was then applied to the cornea after instilling local anesthetic drops (benoxinate 0.5% eye drops) through a sterile setting in the outpatient clinic (first application). The steps of application of Omnilenz® are demonstrated in supplemental video no. 1. AM usually dissolve under the lens within 1 to 2 weeks, and the lens was then removed or replaced (second application) according to the response of healing.

Patients were followed up for at least 1 month and examined everyday until the end of the first week, then at days 14 and 30 of the application of the Omnilenz. Primary outcome measures include epithelial healing and limbal ischemia resolution. Secondary outcomes include BCVA, tolerance to the contact lens (discomfort or need for an eye patch), and complications if any.

## Statistical analysis

Statistical analysis was done using SPSS 11.0 (SPSS Inc., Chicago, IL). The Shapiro-Wilk test was used to test normality distribution of the data. The chi-square test was used to compare nominal data. Paired *t*-test was used to compare parametric data. Significance was set at *p* value ≤ 0.05.

## Results

### Demographic and baseline examination

The study included 23 eyes of 21 patients, with an almost equal presentation of males and females, mean age = 30.3 ± 11.7 years, 2 patients with bilateral injury, and 12 (52.2%) right eye presentation. Alcohol was the most causative agent responsible for 34.8% of the cases, followed by alkaline corrosives (30.4%). All included patients were presented to the casualty department within a few hours after injury. The demographics for all 21 patients (23 eyes) are summarized in Table 1.

**Table 1:** Demographic and baseline examination results of the included patients

Demographic data	Number of eyes (%)
Age (years) mean ± SD	30.3 ± 11.7
Unilateral/bilateral	19/2
Female/male	10/11
Agent	
Alcohol	8 (34.8%)
Alkali	7 (30.4%)
Acid	4 (17.4%)
Unknown	4 (17.4%)
Roper-hall classification (no of eyes)	
I	9 (39.1%)
II	4 (17.4%)
III	6 (26.1%)
IV	4 (17.4%)
Visual acuity	
1. 20/60 or better	5 (21.7%)
2. > CF to < 20/60	10 (43.5%)
3. ≤CF	8 (34.8%)
BCVA log MAR (mean± SD) (min–max)	1.13 ± 0.8 (2.3–0.18)
Epithelial defect	
1. <25%	1 (4.3%)
2. 25–50%	8 (34.8%)
3. 50–75%	4 (17.4%)
4. >75% Limbal ischemia 10 (43.5%)	
1. Mild < 1/3 limbus	13 (56.5%)
2. Moderate (1/3-1/2 limbus)	6 (26.1%)
3. Severe (> 1/2 of limbus)	4 (17.4%)

*SD*, standard deviation; *CF*, counting fingers; *min*, minimum; *max*, maximum; *BCVA*, best-corrected visual acuity; *log MAR*, logarithm of minimum angle of resolution

### Outcome of Omnilenz

#### First application

Omnilenz was performed within the first 2 days following the chemical burn injury (range 0–2). Following the first application of the membrane, the size of the epithelial defect showed a statistically significant reduction (*p* = 0.016). Complete epithelial healing was achieved in 12 eyes (52.2%). There was a statistically significant improvement in the BCVA (mean ± SD log MAR= 0.57±0.7 (range 2.0–0.0), (*p* < 0.001). More than half of the eyes attained visual acuity better than 20/60 (56.5%). Restoration of the limbal vascularity was noticed in 56.5% of the eyes. The well-tolerated lens was reported in 60.9% of the patients with only 8.7% of the eyes requiring patching.

#### Second application

Repeated application of Omnilenz was required in 5 eyes (21.7%). One eye after acidic injury, 3 eyes were caused by alkaline corrosives, and the last one was with an unknown agent. All of which had an ocular chemical burn of grade IV in the Roper-Hall classifications. The size of the epithelial defect was reduced after the second application (*p* = 0.504) with improved BCVA (1.06 ± 0.7 (2.0–0.3), *p* = 0.185) without reaching a statistically significant difference.

#### At the end of the follow-up

**Fig. 1:**
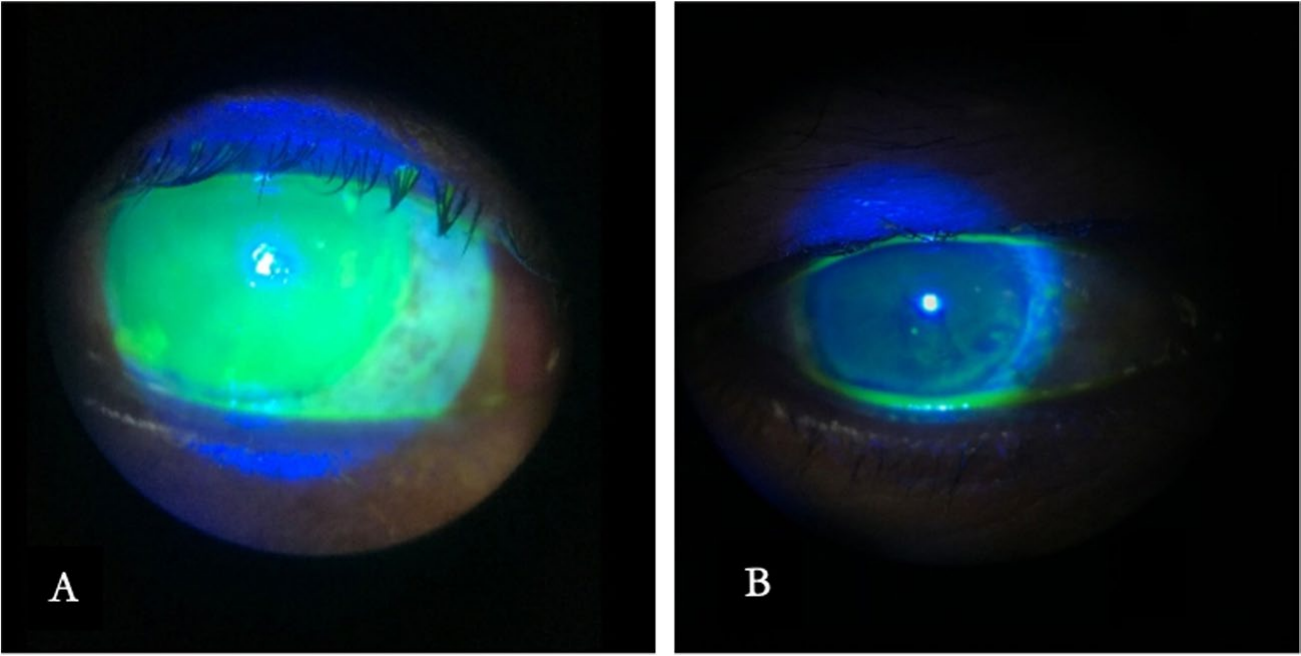
A 31-year-old male patient after alkali corrosive. **A** At presentation. **B** 1 week after Omnilenz application

**Fig. 2:**
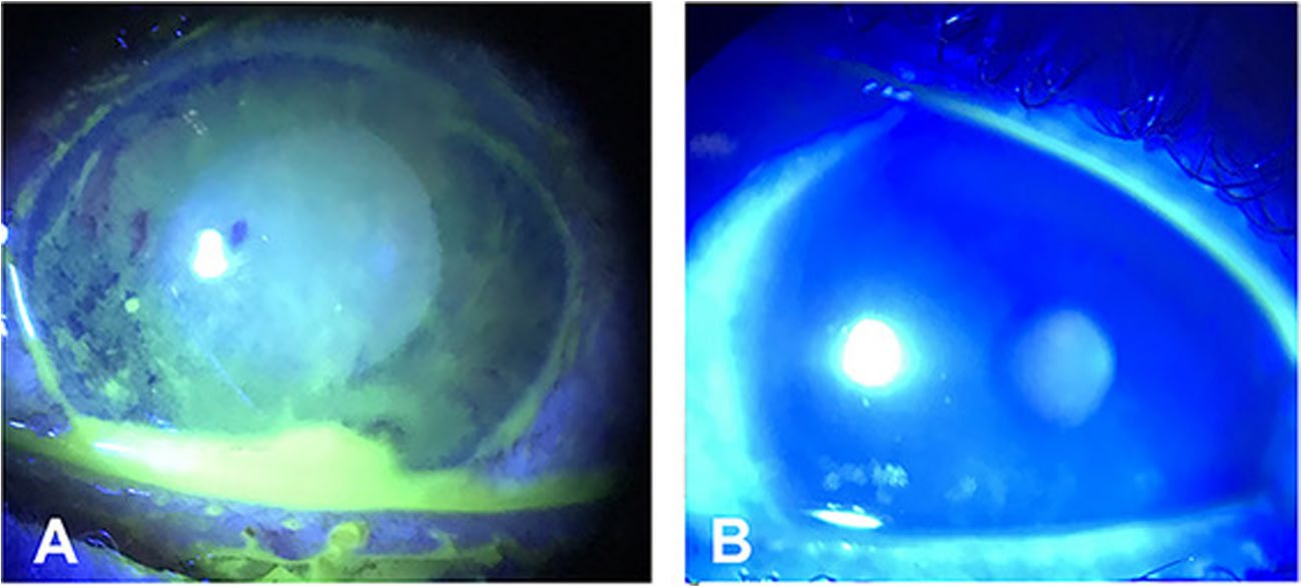
A 35-year-old male patient after acidic corrosive. **A** At presentation. **B** 1 week after Omnilenz application

**Fig. 3:**
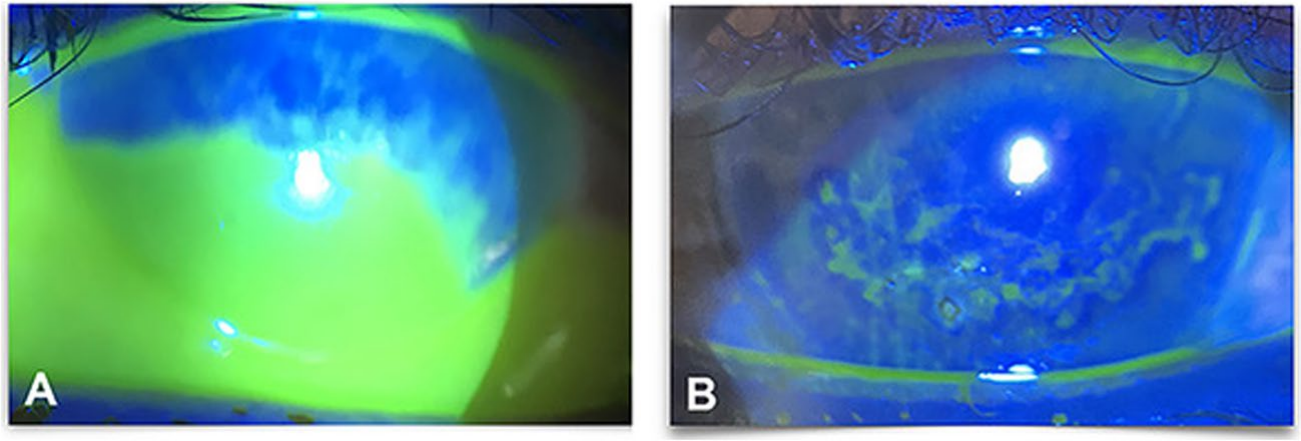
A 43-year-old female patient after alkali corrosive. **A** At presentation. **B** 1 week after Omnilenz application

**Fig. 4:**
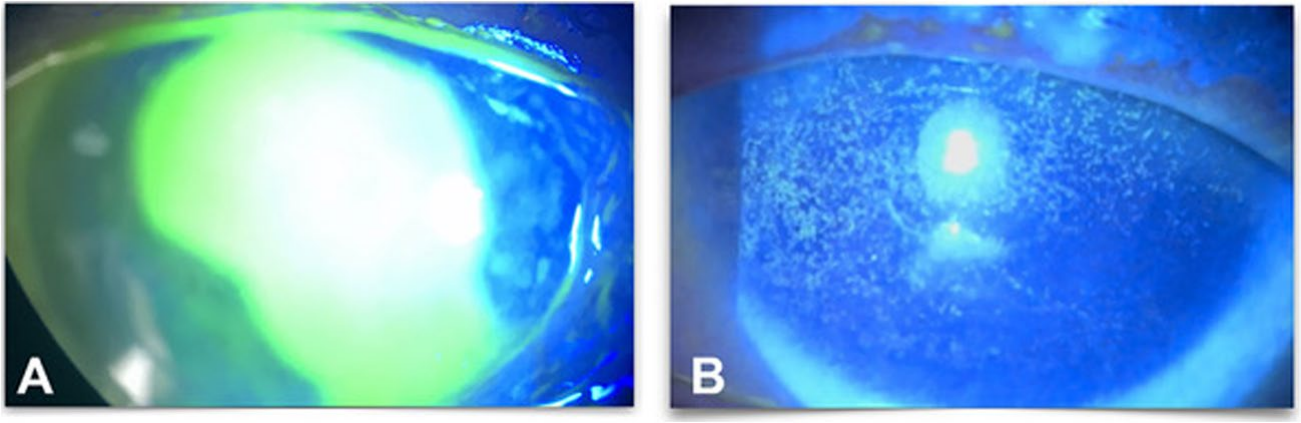
A 28-year-old female patient after alcohol. **A** At presentation. **B** 1 week after Omnilenz application

Complete healing of epithelial defect was obtained in all the eyes. Mild limbal ischemia persists in 3 eyes (13%). Final BCVA showed statistically significant improvement compared to baseline values (*p* < 0.001). None of the eyes required more than 2 applications. The AM dissolved within 7 days following each application. None of the treated eyes developed remarkable complications related to the Omnilenz procedure. None of the eyes required the removal of the lens due to discomfort. Central scarring developed in one case (4.3%), and a temporary rise in intraocular pressure (IOP) occurred in 3 patients (13.1%), all post alkaline corrosive. The IOP in all the 3 cases was controlled medically. Table 2 summarizes the clinical finding following each application of Omnilenz and at the end of the follow-up. Table 3 shows the spreadsheet of clinical findings of the included cases post-treatment. Figs. 1, 2, 3, and 4 show different cases at the presentation and at the end of the follow-up period.

**Table 2:** Clinical finding after Omnilenz along the follow-up period

Parameter	No of eyes (%)		
	1^st^ application	2^nd^ application	At 1 month
	Total = 23	Total = 5	Total = 23
Visual acuity			
1. 20/60 or better	13 (56.5%)	2 (40%)	15 (65.3%)
2. > CF to < 20/60	7 (30.4%)	2 (40%)	7 (30.4%)
3. ≤CF	3 (13.1%)	1 (20%)	1 (4.3%)
Epithelial Defect			
1. Complete healing	12 (52.2%)	1 (20%)	23 (100%)
2. <25%	4 (17.4%)	2 (40%)	0
3. 25-50%	5 (21.7%)	2 (40%)	0
4. 50-75%	2 (8.7%)	0	0
Limbal ischemia			
1. No	13 (56.5%)	2 (40%)	20 (86.9%)
2. Mild	8 (34.8%)	3 (60%)	3 (13.1%)
3. Moderate	1 (4.3%)	0	0
4. Severe	1 (4.3%)	0	0
Tolerance			
1. Tolerated	14 (60.9%)	2 (40%)	–
2. Discomfort	7 (30.4%)	1 (20%)	–
3. Needs patching	2 (8.7%)	2 (40%)	–
Post-healing corneal state			
1. Clear cornea	14 (60.9%)	1 (20%)	19 (82.6%)
2. Corneal edema	8 (34.8%)	3 (60%)	3 (13.1%)
3. Central scarring	1 (4.3%)	1 (20%)	1 (4.3%)

*SD*, standard deviation; *CF*, counting fingers; *BCVA*, best corrected visual acuity; *min*, minimum; *max*, maximum; *log MAR*, logarithm of the minimum angle of resolution

**Table 3:** Spreadsheet of clinical findings of the included cases posttreatment

Eyes	BCVA			Epithelial defect			Limbal ischemia		
	1st	2nd	1 m	1^st^	2^nd^	1 m	1^st^	2^nd^	1 m
1	CF30cm	CF4m	CF4m	4	2	0	3	2	1
2	20/25	–	20/25	0	–	0	0	–	0
3	20/20	–	20/20	0	–	0	0	–	0
4	20/100	–	20/100	2	–	0	2	–	1
5	Cf2m	–	CF2m	3	–	0	3	–	0
6	20/80	–	20/80	1	–	0	1	–	0
7	20/30	–	20/30	0	–	0	0	–	0
8	20/20	–	20/20	0	–	0	0	–	0
9	20/25	–	20/25	0	–	0	0	–	0
10	CF 2m	20/150	20/150	2	2	0	1	1	1
11	20/20	–	20/20	0	–	0	0	–	0
12	20/25	–	20/25	0	–	0	0	–	0
13	20/20	–	20/20	0	–	0	0	–	0
14	20/150	20/40	20/40	2	0	0	2	0	0
15	20/20	–	20/20	0	–	0	0	–	0
16	20/200	20/150	20/150	2	1	0	2	0	0
17	20/40	–	20/40	1	–	0	0	–	0
18	20/25	–	20/25	0	–	0	0	–	0
19	20/80	–	20/80	1	–	0	1	–	0
20	20/25	–	20/25	0	–	0	0	–	0
21	20/150	20/40	20/40	1	0	0	1	–	0
22	20/25	–	20/25	0	–	0	0	–	0
23	20/200	–	20/200	2	–	0	2	–	0

Size of epithelial defect; 0, complete healing; 1, <25%; 2, 25–50%; 3, 50–75%; 4, >75%

Limbal ischemia; 0, complete restoration of vascularity; 1, mild; 2, moderate; 3, severe

*BCVA*, best corrected visual acuity; *CF*, counting fingers; *1*^*st*^, first application; *2*^*nd*^, second application; *1 m*, one month

Results were analyzed for alcohol, alkali, and acid injuries separately. There were 8 patients in the alcohol group; none of them required a second application of AM. All of them attained visual acuity > 20/60 and achieved complete epithelial healing with a resolution of the limbal ischemia by the end of the follow up. Three out of seven eyes (43%) in the alkali group received a second Omnilenz. Only one eye (14%) reaches BCVA > 20/60 in the alkali group. Outcomes in the acidic group were more promising with VA > 20/60 in almost all the eyes (75%). One eye (25%) had a second Omnilenz in the latter group. Four patients had an unknown source of chemical injury, of which 3 patients recovered after the first AMT, and for one eye, it failed and required a second AMT.

A comparison between grades I and II vs grades III and IV (Roper-Hall classification) with respect to clinical response was carried out. Following the 1^st^ application; there was a statistically significant difference between the 2 groups regarding the size of the epithelial effect (*p* = 0.01), the limbal ischemia (*p* = 0.000), and the tolerance to the lens (*p* = 0.00). At the end of the follow up, there was no statistical difference between the 2 groups (Table 4).

**Table 4:** Clinical comparison among the studied eyes according to the Roper-Hall classification following 1^st^ application

After 1^st^ application	No. of eyes (%)		*P* value (chi-square)
	Roper Hall I and II	Roper Hall III and IV	
Size of epithelial defect			
Complete healing	12	1	
<25%	1	3	0.01*
25–50%	0	5	
50–75%	0	1	
>75%	0	0	
Limbal ischemia			0.000*
no	13	0	
mild	0	4	
moderate	0	4	
severe	0	2	
Tolerance			0.000*
Tolerant	13	1	
Discomfort	0	8	
Need patching	0	1	
Total	13	10	–

*Statistically significant value

## Discussion

Omnigen (OG) is a low-temperature vacuum-dehydrated amniotic membrane transplant (AMT). Dehydrated AMs are one of the methods for AM preservation that can be stored at room temperature, rendering AMs more feasible for transportation and preservation. Dehydrated AM only require a small droplet of balanced salt solution to be rehydrated before manipulation. In contrast, cryopreserved AMs must be stored in the frozen state which is more expensive during transportation. Omnilenz-Omnigen complex is placed onto the ocular surface, without any need for suturing or gluing. Moreover, its application can be repeated. The AM usually lasts for a few days (usually around 7 days).

In the current study, we evaluate the use of Omnilenz in 23 eyes with acute chemical eye injury mostly after alcohol and alkaline exposure. Complete epithelial healing with the restoration of limbal vascularity was achieved in more than half of the eyes after the first application. Five eyes required a second application. All patients were followed up for at least one month. We did not encounter special complications related to the Omnilenz itself in any of our patients.

In our study, Omnilenz was applied within 2 days after the injury. Encouraging results of immediate application have been demonstrated by various studies [8, 9]. Such early intervention was rendered feasible, mostly because of the easiness of application of Omnilenz in the clinic. Prabhasawat et al. [10] showed that AMT performed within 5 days in eyes with grades II and III CEI resulted in earlier epithelial healing and less limbal stem cell deficiency (LSCD) than AM later.

In addition to the lapse in time after chemical injury, the severity of the injury is another factor that seems to affect the final outcome. It has been shown that eyes with a low-grade chemical burn gain benefit from AMT while this influence could not be achieved in eyes with severe injuries [11]. In this context, we found that Omnilenz was not effective in the restoration of ocular surface integrity in eyes with severe burns (grade IV in the Roper-Hall classification). In agreement, Meller et al. [12] analyzed the clinical outcome of AMT in patients with acute CEI. They concluded that in severe cases, AMT could not restore the limbal cell population. This limitation has been reported also in another published study [13].

In etiological perspective, alcohol was the commonest causative agent in our series owing to the era of COVID-19 with lots of people using alcohol for hand sterilization and accidental eye injury. Concerning the groups, there were differences in the outcomes. Obviously, the results were more favorable in patients with alcohol injury than alkali injury. Statistical analysis was not carried out owing to the small number in each group. Yet, the outcomes were least favorable in the alkali group. On the other hand, Tejwani et al. [14] reported different results following the use of cryopreserved AM. In their series, alkali injuries had a better prognosis with an overall success rate higher than acid injury.

Mehta et al. [15], in a recent retrospective study, studied the application of Omnilenz in eyes with CEI, 2 eyes with acute and 15 with chronic affection. They reported favorable outcomes in terms of the restoration of BCVA.

Even though limitations do exist as the lack of a control group with AMT with suture, the current study is the first prospective study highlighting the role of Omnilenz in acute CEI. Future large-scale trials with longer follow-up periods will be vital to elucidate more the possible benefits, restrictions, and the perfect timing as well as the grade of chemical burn ideal for the application of the Omnilenz.

In conclusion, Omnilenz proved to be easy to apply in an outpatient clinic setting, reasonably well tolerated by patients, with promising success. Omnilenz might be included in the paradigm of early management of acute ocular chemical burn. The grade of ocular burn on presentation and timing of intervention with AMT were important factors that influence the outcomes.

### Supplementary information

ESM 1 (MOV 55948 kb)
